# Accidente ofídico en el departamento de Nariño, Colombia: análisis retrospectivo, 2008-2017

**DOI:** 10.7705/biomedica.4830

**Published:** 2019-12-30

**Authors:** María José Sevilla-Sánchez, Diana Mora-Obando, Jhon Jairo Calderón, Jimmy Alexander Guerrero-Vargas, Santiago Ayerbe-González

**Affiliations:** 1 Facultad de Ciencias Exactas y Naturales, Departamento de Biología, Grupo de Investigación en Ecología Evolutiva, Universidad de Nariño, Pasto, Colombia Universidad de Nariño Departamento de Biología Grupo de Investigación en Ecología Evolutiva Universidad de Nariño Pasto Colombia; 2 Laboratorio de Venómica Evolutiva y Traslacional, Universidad de Valencia, Valencia, España Laboratorio de Venómica Evolutiva y Traslacional Universidad de Valencia Valencia España; 3 Grupo de Investigaciones Herpetológicas y Toxinológicas, Centro de Investigaciones Biomédicas-Bioterio, Departamento de Biología, Facultad de Ciencias Naturales, Exactas y de la Educación, Universidad del Cauca, Popayán, Colombia Universidad del Cauca Departamento de Biología Facultad de Ciencias Naturales, Exactas y de la Educación Universidad del Cauca Popayán Colombia

**Keywords:** Bothrops, mordeduras de serpientes, enfermedades desatendidas, Colombia, Bothrops, snake bites, neglected diseases, Colombia

## Abstract

**Introducción.:**

El ofidismo es un relevante problema de salud pública y, en Colombia, se incluyó como un evento de notificación obligatoria desde el año 2004. Por ser un país tropical con gran diversidad ecosistémica, ocupa el tercer puesto en Latinoamérica, después de México y Brasil, en presentar el mayor número de accidentes ofídicos.

**Objetivo.:**

Realizar un análisis retrospectivo del accidente ofídico en el departamento de Nariño, con base en los eventos notificados entre los años 2008 y 2017 al Instituto Departamental de Salud de Nariño y al Sistema de Vigilancia en Salud Pública de Colombia.

**Materiales y métodos.:**

Se hizo un análisis de tipo descriptivo y retrospectivo a partir de la recopilación e interpretación de la información consignada en las fichas de notificación para accidente ofídico del Instituto Departamental de Salud de Nariño, entre los años 2008 y 2017. Se representó la frecuencia del accidente ofídico a nivel municipal mediante la elaboración de un mapa y se identificaron los géneros responsables del mismo.

**Resultados.:**

Se reporta un total de 1.110 casos. El 78,13 % de los municipios hizo alguna notificación. Se observa un patrón de aumento constante en el número de casos durante los 10 años, a excepción de 2017. Las características sociodemográficas se mantuvieron durante el periodo de estudio.

**Conclusiones.:**

El municipio de San Andrés de Tumaco, el sexo masculino y las áreas rurales son los principales afectados por el ofidismo causado, en mayor medida, por el género *Bothrops*. La mayor incidencia se presentó en el mes de julio.

## Abstract (en)

**Introduction.:**

Snakebite envenoming is a relevant public health problem, and, in Colombia, it was included as a mandatory notification event since 2004. Because it is a tropical country with great ecosystem diversity, it occupies third place in Latin America, after Mexico and Brazil, reaching the highest number of snakebites.

**Objective.:**

To carry out a retrospective analysis of snakebites in the department of Nariño based on the notifications reported to the *Instituto Departamental de Salud de Nariño* and the *Sistema de Vigilancia en Salud Pública de Colombia* between the years 2008 and 2017.

**Materials and methods.:**

A descriptive and retrospective analysis was carried out based on the study, and interpretation of the information contained in the notification sheets for ophidian accidents of the *Instituto Departamental de Salud de Nariño* between the years 2008 and 2017. The snakebite frequency at the municipal level was represented by the elaboration of a map and the responsible genus were identified.

**Results.:**

A total of 1,110 cases were reported for ophidian accidents. Seventy- eight point thirteen per cent of the municipalities made some notification. A pattern of constant increase in the case number during the 10 years is evident, with exception of 2017; the sociodemographic characteristics are maintained.

**Conclusions.:**

The municipality of San Andrés de Tumaco, the masculine gender and the rural areas are mostly affected by snakebites, caused mainly by the *Bothrops* genus and the highest snakebite incidence was seen in July.

Las serpientes pertenecen a la clase Reptilia, orden Squamata y suborden Serpentes ^1^. Se distribuyen en todos los continentes y son animales con una gran capacidad de adaptación, los cuales habitan diversos nichos, pisos térmicos y ecosistemas ^1^^,^^2^. Tienen un rol fundamental en la dinámica normal de las comunidades biológicas, regulando la densidad poblacional de sus presas, como lombrices, caracoles, insectos, peces, anfibios, reptiles, aves y mamíferos ^3^.

Algunas familias de serpientes han desarrollado la capacidad de producir veneno en glándulas salivales modificadas, para su defensa y alimentación, pues inmovilizan, causan la muerte y digieren a sus presas ^4^. Los venenos de origen animal son mezclas biológicas complejas de sales, lípidos, carbohidratos, péptidos de masa molecular baja y proteínas con actividades enzimáticas o sin ellas ^5^. La diversa gama de componentes incluyen: neurotoxinas, proteasas de serina, metaloproteasas de clases P-I, P-II y P-III, lectinas de tipo C, proteínas séricas ricas en cisteína, convulxina, fosfolipasas A2 miotóxicas (D49 y K49), fosfolipasas B, hialuronidasas, fosfodiesterasas, factores de crecimiento neural e inhibidores de proteasas de serina, entre otros ^6^^,^^7^. Estos componentes pueden variar dependiendo de la familia, el género, la especie, la distribución geográfica y el estado de madurez de la serpiente (variación ontogénica), y contribuyen directamente con los síntomas fisiopatológicos, locales, sistémicos o ambos, observados durante el envenenamiento ocasionado por las especies de importancia clínica ^8^^,^^9^.

A nivel mundial, se han reportado 3.567 especies de serpientes, distribuidas en 465 géneros y 20 a 30 familias ^10^^,^^11^. En Colombia, se registran aproximadamente 309 especies de serpientes agrupadas en 8 familias, las cuales se distribuyen en todo el territorio nacional, desde los cero hasta los 3.500 msnm ^3^. Las familias Viperidae y Elapidae son las de mayor importancia clínica, con 19 y 30 especies, respectivamente ^1^^,^^12^. Las principales especies responsables de los accidentes ofídicos son las de los géneros *Bothriechis* , *Bothriopsis* , *Bothrocophias* , *Bothrops* , *Crotalus* , *Lachesis* y *Porthidium*, entre los vipéridos, y *Micrurus* e *Hydrophis* , entre los elápidos ^1^^,^^11^^,^^13^^,^^14^. Sin embargo, entre los colúbridos, en las subfamilias Colubrinae y Dipsadinae ^14^, se encuentran especies de posible importancia en salud pública, pertenecientes a los géneros *Apostolepis* , *Helicops* , *Philodryas* , *Thamnodynastes* y *Xenodon*, todas distribuidas por debajo de los 1.000 msnm ^3^.

El accidente ofídico u ofidismo es el cuadro clínico desencadenado por las mordeduras de las serpientes, ya sean venenosas o no, con inoculación de veneno o sin ella ^12^; en la actualidad, se reconoce como un relevante problema de salud pública en muchos países del mundo, especialmente los latinoamericanos ^6^^,^^15^. Mundialmente, se estima que cerca de cinco millones de personas han sido víctimas de accidentes por ofidios venenosos o no venenosos, cuya intoxicación o envenenamiento puede resultar en alteraciones fisiopatológicas, locales o sistémicas, con graves secuelas sociales y económicas ^16^. Esta cifra podría estar subestimada, ya que los pacientes no alcanzan a ser atendidos en el centro de salud, debido a la distancia a la que se encuentran o porque se niegan a recibir un tratamiento médico ^15^^,^^17^^,^^18^.

En Colombia, el ofidismo se incluyó como un evento de notificación obligatoria por el Sistema Nacional de Vigilancia en Salud Pública (Sivigila) desde octubre de 2004, en la Circular 092 del Ministerio de Salud; pero solo hasta el año 2007, los casos comenzaron a notificarse de manera constante, lo cual ha permitido una mejor aproximación al número de registros del ofidismo ^4^^,^^18^^,^^19^.

Colombia, al ser un país tropical de temperaturas cálidas y templadas, con gran diversidad ecosistémica ^3^^,^^20^^,^^21^, es el tercero en Latinoamérica, después de México y Brasil, en presentar el mayor número de casos de accidentes ofídicos ^2^^).^ estos accidentes disminuyen con las bajas temperaturas y el aumento de altitud debido a la reducción de la riqueza de ofidios ^3^. Por lo tanto, en la región Andina, por encima de los 1.500 msnm, se registran dos o tres especies de vipéridos y tres a cuatro especies de corales, mientras que, en las regiones de la Amazonia y el Chocó biogeográfico, se pueden encontrar hasta 45 especies de serpientes ^22^

Los estudios epidemiológicos disponibles muestran que, en nuestro país, se presentan entre 2.000 y 4.500 accidentes cada año, con una incidencia que varía entre 6,2 casos por 100.000 habitantes, en las regiones menos pobladas, a 20 casos por 100.000 habitantes, en las zonas más densamente habitadas, y una mortalidad que oscila igualmente entre 0,04 y 7,6 %, según las diferentes regiones del país ^23^.

Para el año 2016, el Instituto Nacional de Salud de Colombia mediante el Sivigila, reportó 4.636 casos, una incidencia de 9,5 casos por cada 100.000 habitantes y una letalidad de 0,4 % ^24^; para el año 2017, se reportaron 4.978 casos con una incidencia de 10,1 casos por cada 100.000 habitantes, lo cual indica un aumento del 6 % respecto al anterior ^25^; y para el año 2018, se reportaron 5.286 casos en el boletín de la semana epidemiológica 52 ^26^; no obstante, está pendiente la publicación del informe final del evento.

Según Gómez (2011), el 31,2 % de los casos por accidente ofídico en Colombia ocurren en la región occidental, el 23,8 %, en la Costa Atlántica, el 18,9 %, en la Orinoquia, el 18,2 %, en la región centro-oriente, y el 7,7 %, en la Amazonia ^27^. Aunque Nariño es catalogado como un departamento con baja frecuencia de accidentes ofídicos ^19^, estas cifras podrían incrementarse considerablemente, ya que muchos de los casos no son reportados a los centros de salud ^28^ y otros son tratados en el departamento del Cauca ^3^^,^^12^; además, es uno de los departamentos donde se ha registrado el mayor número de defunciones ^24^^,^^25^. Es evidente que, pese a la existencia de registros nacionales, factores como la falta de acceso a los servicios de salud‚ problemas con el diligenciamiento‚ flujo y reporte de la información, e incluso, el tratamiento por métodos tradicionales alternativos‚ conducen a un preocupante subregistro ^18^.

El departamento de Nariño incluye tres de las siete regiones del país, Pacífica, Andina y Amazónica, las cuales albergan un total de 239 especies de serpientes ^22^. Según los registros de la Colección Herpetológica del Museo de Historia Natural PSO-CZ de la Universidad de Nariño, en el departamento se encuentran especies de los géneros *Boa*, *Atractus*, *Chironius*, *Clelia*, *Dipsas*, *Erythrolamprus*, *Mastigodryas*, *Sibon*, *Imantodes*, *Leptodeira*, *Liophis*, *Spilotes*, *Tantilla*, *Helicops*, *Bothrocophias*, *Bothrops, Lachesis*, *Bothriopsis*, *Micrurus*, entre otros, agrupados en las familias Boidae, Colubridae, Leptotyplopidae, Elapidae y Viperidae.

Las características ecológicas tanto de la franja del Chocó biogeográfico como del piedemonte costero del Pacífico, los Andes del norte y las estribaciones superiores de la Amazonia, le confieren a Nariño una gran riqueza biológica con representación, prácticamente, de todos los ecosistemas (alta y media montaña, bosques secos, humedales, etc.) ^21^ y, con ello, una gran diversidad de serpientes, lo cual incrementa el riesgo de envenenamientos.

Hasta la fecha, no se han realizado trabajos que permitan dimensionar el impacto del accidente ofídico en la región y reconocer los géneros, e incluso especies, de las serpientes responsables de los mismos. Por lo tanto, a partir de los registros del Instituto Departamental de Salud de Nariño y del Sivigila, esta investigación describe retrospectivamente la frecuencia y la incidencia de los casos por mordedura de serpiente en una década (2008- 2017), teniendo en cuenta variables como la actividad desempeñada durante el accidente, la pertenencia étnica, la edad, las características relacionadas con el agente causal y la sintomatología, entre otras. Además, determina los géneros de serpientes responsables y se referencian geográficamente en relación con los casos ocurridos en un periodo de 10 años.

## Materiales y métodos

### Área de estudio

**Figura 1.**
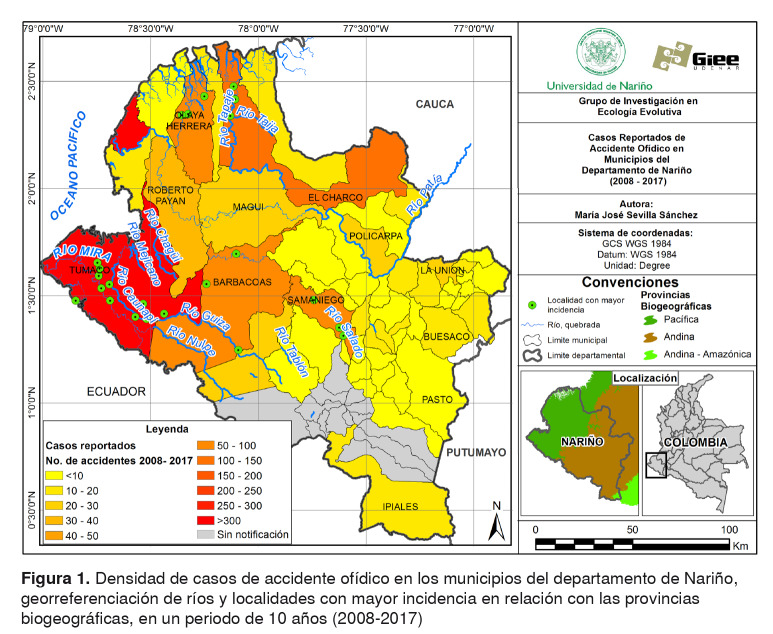
Densidad de casos de accidente ofídico en los municipios del departamento de Nariño, georreferenciación de ríos y localidades con mayor incidencia en relación con las provincias biogeográficas, en un periodo de 10 años (2008-2017)

El departamento de Nariño, localizado al suroccidente de Colombia, entre los 00°31’08” y los 02°41’08” de latitud norte, y los 76°51’19” y 79°01’34” de longitud oeste (figura 1), está conformado por 64 municipios. Limita al norte con el departamento del Cauca; al sur, con la República de Ecuador; al oriente, con los departamentos de Putumayo y Cauca, y al occidente, con el océano Pacífico ^21^. Tiene una extensión de 33.268 km^2^ , aproximadamente, el 3 % del territorio colombiano, con una topografía estructurada principalmente por los Andes, valles interandinos, llanuras y piedemontes, tanto en el Pacífico como hacia la Amazonia ^20^^,^^29^.

Como producto de esta accidentalidad orográfica, se diferencian tres provincias biogeográficas: Pacífica, Andina y Andina-Amazónica. La provincia del Pacífico (o Chocó) corresponde a la llanura pacífica y el piedemonte occidental de la cordillera Occidental, la provincia Andina está representada por el macizo andino y la provincia Andina-Amazónica abarca el piedemonte amazónico de la cordillera centro-oriental ^21^.

### Población y muestra

Se analizaron los casos de accidente ofídico notificados al Sivigila por los 64 municipios del departamento de Nariño, durante el periodo comprendido entre 2008 y 2017.

Como criterios de inclusión, se tuvieron en cuenta todos los casos de pacientes nacionales y extranjeros ocurridos en el departamento de Nariño y que fueron confirmados por la entidad de salud como accidente ofídico: pacientes con signos y síntomas o sin ellos, mordidos por una serpiente, identificada o no. Se excluyeron los casos atendidos en Nariño correspondientes a otros departamentos (por ejemplo, Putumayo y Cauca), los casos reportados por otros departamentos ocurridos en Nariño y los datos duplicados ^2^.

### Técnicas de recolección y análisis de la información

Se hizo un análisis de tipo descriptivo y retrospectivo a partir de la recopilación e interpretación de la información (básica y complementaria) consignada en las fichas de notificación epidemiológica para accidente ofídico (código INS: 100) del Instituto Departamental de Salud de Nariño, y las bases de datos (previa autorización de la dirección), así como de los boletines semanales e informes mensuales y anuales, disponibles en la página web del Sivigila del Instituto Nacional de Salud ^30^.

Las variables de la ficha de notificación analizadas fueron ^31^:

1. Condiciones sociodemográficas del paciente: semana epidemiológica en la que ocurrió el accidente, edad y sexo del paciente, municipio de residencia, zona y cobertura de seguridad social.
2. Notificación del accidente: clasificación inicial del caso, hospitalización y condición final del paciente.
3. Caracterización del accidente: mes de ocurrencia del accidente, actividad realizada al momento del accidente, tipo de atención inicial, prácticas no médicas, localización de la mordedura, género, especie y nombre común de la serpiente, este último, en caso de que el género no hubiera sido registrado.
4. Manifestaciones del accidente y sus complicaciones: signos locales (marcas de dientes o colmillos, edema, sangrado, flictenas, equimosis); síntomas locales (dolor, disestesia, parestesia); complicaciones locales (sobreinfección, necrosis, amputación) y sistémicas (hemorragia sistémica, parálisis respiratoria, falla renal, cardíaca o multisistémica), y gravedad del accidente (sin envenenamiento, leve, moderado o grave).
5. Atención del accidente: uso de suero antiofídico, tiempo, tipo de suero empleado, dosis usada de suero, duración del tratamiento con suero antiofídico

Los datos se analizaron estadísticamente empleando medidas de tendencia central para variables cuantitativas, frecuencias (relativas y absolutas) y porcentajes para variables cualitativas en el programa Excel™, y se representaron gráficamente en el programa Bioestat™, versión 5.3 ^2^. La incidencia por año y mes fue calculada como el cociente entre el número de casos y la población correspondiente a los años 2008 a 2017, según las proyecciones demográficas del censo del DANE del 2005 para el departamento de Nariño ^32^.

Se integró la información registrada en las fichas con la información suministrada por el Museo de Historia Natural PSO-CZ, las bases de datos relacionadas (*Global Biodiversity Information Facility*, GBIF) y el sistema de información sobre biodiversidad de Colombia, SIBColombia) ^33^^,^^34^, con el objetivo de georreferenciar los géneros y especies de interés clínico.

Los mapas fueron elaborados con el *software* QGIS™, 3.0.1, Girona de 1991, utilizando las planchas de Colombia, el departamento de Nariño y los municipios del departamento de Nariño, del Instituto Geográfico Agustín Codazzi ^35^. Se levantó un mapa de densidad con el programa arcGIS™, versión 10.1, para representar la frecuencia del accidente ofídico en los municipios, e identificar las localidades y provincias biogeográficas con mayor número de casos notificados.

El análisis de estas variables permitió establecer la relación entre municipio, localización geográfica del accidente, género de la serpiente al cual se atribuye el accidente y su distribución geográfica en el departamento de Nariño.

Además, teniendo en cuenta que los cuadros clínicos desencadenados por envenenamiento botrópico, lachésico, crotálico, elapídico o colúbrico son diferenciales, se contrastaron los síntomas locales y sistémicos de cada paciente con las manifestaciones y complicaciones descritas por Ayerbe (2009) para cada tipo de envenenamiento ^12^. Este análisis permitió verificar la concordancia entre la serpiente causante del ofidismo y el cuadro clínico, y detectar el posible género de serpiente responsable del envenenamiento cuando la especie no fue identificada.

### Consideraciones éticas

Se garantizó la confidencialidad de la información, bajo la Ley 1273 del 2009 y 1266 del mismo año.

## Resultados

### Frecuencia e incidencia de los accidentes ofídicos en el departamento de Nariño

Se analizaron 1.110 accidentes ofídicos durante un periodo de 10 años, a partir de los cuales de determinó un promedio de 111 casos por año, una incidencia anual de 6,54 casos por cada 100.000 habitantes y 27 defunciones. El 78,13 % de los municipios notificó al Sivigila los casos de ofidismo ocurridos durante los años 2008 a 2017 (figura 1) y se resalta que los pacientes tardan en promedio 4,48 días, con un máximo de hasta 120 días, en notificar el accidente a un centro de salud. Los municipios más afectados por esta enfermedad tropical desatendida, son aquellos que hacen parte de la provincia biogeográfica Pacífica, donde se registró el 78,47 % de todos los casos. Los municipios ubicados dentro de la Provincia Andina y la Andino-Amazónica presentaron el menor número de casos (19,48 %) (figura 1, cuadro 1).

**Cuadro 1:** Número de casos de accidente ofídico en los municipios del departamento de Nariño con relación a las provincias biogeográficas, en un periodo de 10 años (2008-2017)

Municipio	n	Provincia biogeográfica
		
San Andrés de Tumaco	311	P
El Charco	133	P
Samaniego	89	P
Barbacoas	75	P
Olaya Herrera (Bocas de Satinga)	57	P
Roberto Payán (San José)	31	P
La Unión	27	A
Policarpa	27	P
Ricaurte	26	P
Magüí (Payán)	21	P
La Tola	20	P
Leiva	19	A
Santa Bárbara (Iscuandé)	17	P
Buesaco	15	AA
El Tablón de Gómez	15	AA
Linares	14	A
Colón (Génova)	12	A
Francisco Pizarro (Salahonda)	12	P
Ipiales	11	AA
La Florida	11	A
San Lorenzo	11	A
Sandoná	11	A
Taminango	11	A
El Rosario	10	P
Mosquera	10	P
Ancuya	9	A
San Pablo	9	A
Cumbitara	8	P
Chachagüí	7	A
Consacá	7	A
El Tambo	7	A
Los Andes (Sotomayor)	8	P
Santacruz (Guachavés)	7	P
Arboleda (Berruecos)	6	A
El Peñol	6	P
San Pedro De Cartago	6	A
Albán (San José)	5	AA
Providencia	4	A
Imués	3	A
Pasto	3	AA
San Bernardo	3	A
Yacuanquer	3	A
Belén	2	A
Guaitarilla	2	A
La Cruz	2	AA
La Llanada	2	P
Tangua	2	A
Funes	1	AA
Mallama (Piedrancha)	1	P
Nariño	1	A
Total		1.110

P: provincia biogeográfica Pacífica; A: provincia biogeográfica Andina; AA: provincia biogeográfica Andino-Amazónica

Los municipios más afectados por esta problemática fueron: San Andrés de Tumaco, donde la mayor frecuencia de notificación se registró en el corregimiento de Llorente y las veredas Candelillas, La Guayacana, Tangareal, Chilví, Imbilí, Caunapí y Zabaleta (caserío Bajo Inda), y el municipio El Charco, con mayor notificación en las veredas Taija, El Hormiguero, El Charco y La Capilla; ambos municipios están ubicados en la llanura pacífica.

El municipio de Samaniego, situado en el piedemonte andino pacífico del departamento, presentó tres localidades con mayor reporte de casos (veredas Cartagena, Piedra Blanca y Betania), sin desconocer el corregimiento de Altaquer, y las veredas Coscorrón de Pumbí y Teraimbe (en Barbacoas) y Bocas de Satinga y Bocas de Prieta (en Olaya Herrera) como localidades con alto número de casos. En estos municipios, la mayoría de los accidentes se registraron en lugares asociados a los ríos Mira, Patía, Güisa, Nulpe, Chagüí, Gualajo, Ispí, Las Juntas, Mexicano, Tablón Salado, Taija, Tapaje y Caunapí.

Los municipios localizados en los complejos paramunos del Nudo de los Pastos por encima de los 3.500 msnm, como Aldana, Contadero, Córdoba, Cuaspud, Cumbal, Guachucal, Gualmatán, Iles, Ospina, Potosí, Puerres, Pupiales, Sapuyes y Túquerres, no reportaron accidentes durante este periodo.

**Figura 2.**
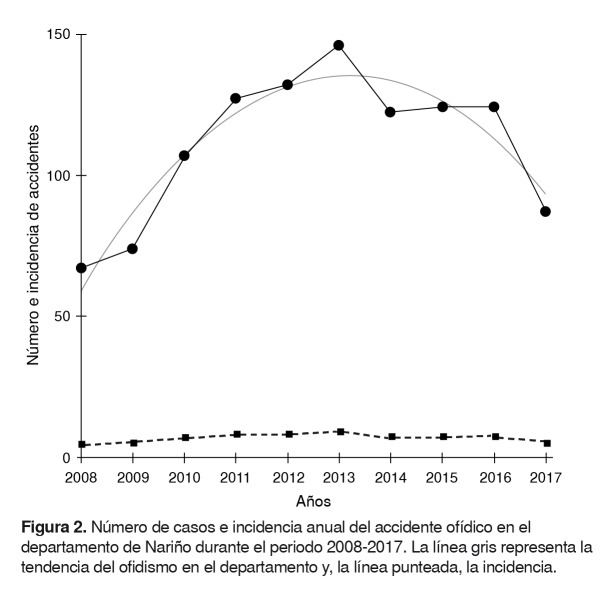
Número de casos e incidencia anual del accidente ofídico en el departamento de Nariño durante el periodo 2008-2017. La línea gris representa la tendencia del ofidismo en el departamento y, la línea punteada, la incidencia.

Con base en los registros del Sivigila para el departamento durante la década de análisis y la incidencia anual, se observa un incremento durante los primeros seis años, pasando de 67 casos en el año 2008 a 146 casos en el 2013. Para los años de 2014 a 2017, se observó un descenso en el número de accidentes de 122 a 87 (figura 2).

**Figura 3.**
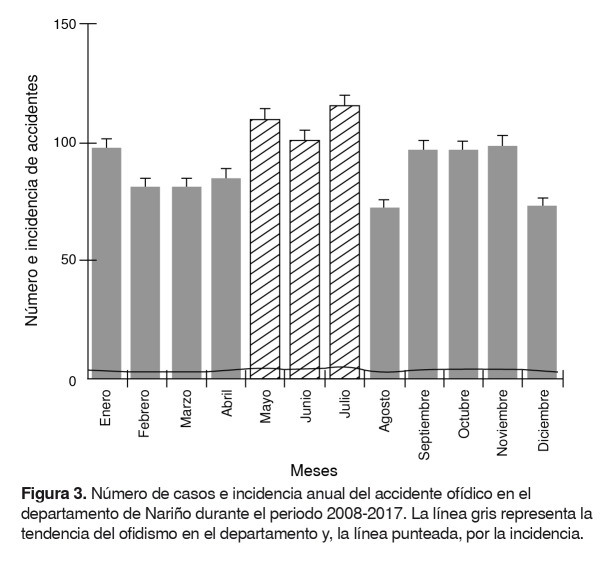
Número de casos e incidencia anual del accidente ofídico en el departamento de Nariño durante el periodo 2008-2017. La línea gris representa la tendencia del ofidismo en el departamento y, la línea punteada, por la incidencia.

La frecuencia del accidente ofídico en el departamento de Nariño, estimada como el número de casos acumulados por mes durante los 10 años y la incidencia mensual, muestran que julio es el mes con mayor número de accidentes, con un total de 116 casos, seguido de mayo con 110 y junio con 101 casos. Los meses cuando se registró el menor número de accidentes fueron agosto con 72 notificaciones, diciembre con 73, y febrero y marzo, cada uno con 81 casos (figura 3).

Respecto al sexo, los accidentes suceden a razón de dos hombres por cada mujer. La media de edad en personas que sufrieron ofidismo fue de 33,12 años (desviación estándar, DE=18), la mediana fue de 30 años, la edad mínima un año y la máxima 84 años. El 34,86 % de los casos se presentó en la población joven activa productivamente; los grupos étnicos más afectados fueron el negro, el mulato y el afrodescendiente, y el mayor número de casos sucedieron cuando las víctimas realizaban actividades agrícolas (cuadro 2).

**Cuadro 2:** Características sociodemográficas del accidente ofídico en el departamento de Nariño, según Sivigila, 2008-2017

Variable	Característica	n	%
			
Sexo	Masculino	780	70,27
	Femenino	329	29,64
	No reporta	1	0,09
Rango de edad (años)	0-15	185	16,67
	16-30	387	34,86
	31-45	249	22,43
	46-60	190	17,12
	61-75	76	6,85
	76 en adelante	23	2,07
Pertenencia étnica	Indígena	66	5,95
	ROM, gitano	3	0,27
	Raizal de San Andrés y Providencia	7	0,63
	Palenquero de San Basilio	1	0,09
	Negro, mulato, afrocolombiano o afrodescendiente	581	52,34
	Ninguna de los anteriores	452	40,72
Área procedencia	Cabecera municipal	149	13,42
	Centro poblado	134	12,07
	Rural / disperso	827	74,50
Tipo de régimen en salud	Contributivo	69	6,22
	Subsidiado	814	73,33
	Excepción	4	0,36
	Especial	15	1,35
	Indeterminado	8	0,72
	No asegurado	200	18,02
Actividad en el momento	Recreación	78	7,03
del accidente	Actividad agrícola	643	57,93
	Oficios domésticos	117	10,54
	Recolección de desechos	7	0,63
	Actividad acuática	23	2,07
	Caminar por senderos abiertos o trocha	138	12,43
	Otro	79	7,12
	No determinado	25	2,25

En el 93,88 % de los pacientes, los accidentes se presentaron en las extremidades superiores e inferiores, variable que podría estar relacionada con la actividad reportada durante el momento del accidente, y en el 78,74 % de los casos, se evidencian marcas de colmillos en la mordedura (cuadro 3).

**Cuadro 3:** Características relacionadas con el agente causal del accidente ofídico, según registros del Sivigila en el departamento de Nariño, 2008-2017

Variable	Característica	n	%
			
Huellas de colmillos	Sí	874	78,74
	No	233	20,99
	No reporta	3	0,27
Serpiente capturada	Sí	364	32,79
	No	744	67,03
	No reporta	2	0,18
Localización de la	Cabeza (cara)	15	1,35
mordedura	Miembros superiores	525	47,30
	Miembros inferiores	517	46,58
	Dedos de la mano	7	0,63
	Dedos de pie y de mano	2	0,18
	Tórax anterior	23	2,07
	Abdomen	6	0,54
	Espalda	7	0,63
	Cuello	2	0,18
	Genitales	1	0,09
	Glúteos	2	0,18
	No reporta	3	0,27
			

### Serpientes responsables del ofidismo

**Figura 4.**
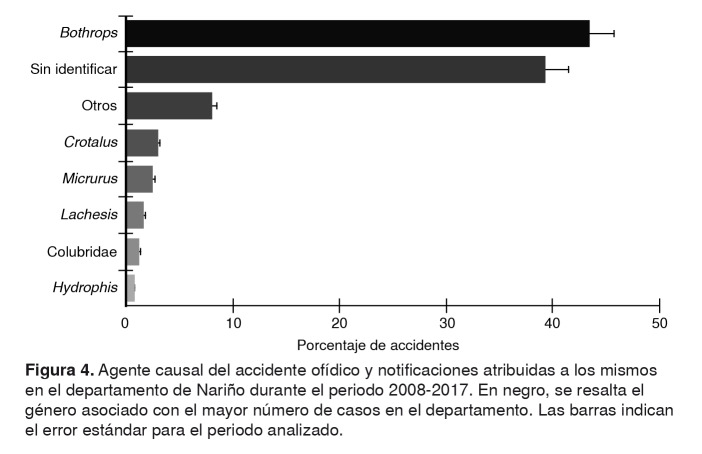
Agente causal del accidente ofídico y notificaciones atribuidas a los mismos en el departamento de Nariño durante el periodo 2008-2017. En negro, se resalta el género asociado con el mayor número de casos en el departamento. Las barras indican el error estándar para el periodo analizado.

Los vipéridos son los responsables del mayor número de accidentes reportados en el departamento, entre los años 2008 y 2017. De un total de 1.110 casos, el 48,02 % fueron causados por esta familia, el 3,24 %, por la familia Elapidae, y el 1,26 %, por la familia Colubridae; para el 47,48 % restante, se desconoce la identificación de la serpiente. Respecto al género de las serpientes, el 43,60 % de los casos fueron atribuidos al género *Bothrops*, 2,88 % a *Crotalus*, 2,52 % a *Micrurus*, 1,53 % a *Lachesis*, 0,72 % a *Hydrophis* y 9,28 % a otros géneros que comprenden serpientes venenosas y no venenosas (figura 4); en el 39,46% de los casos, se omitió esta información.

**Figura 5.**
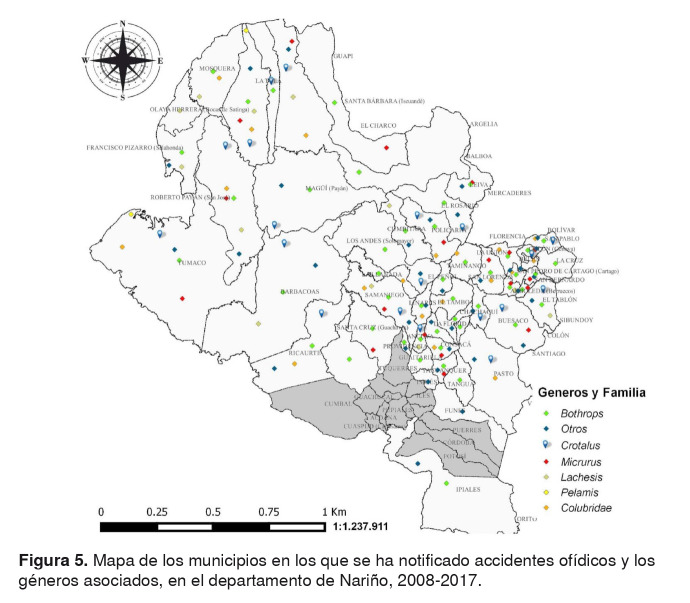
Mapa de los municipios en los que se ha notificado accidentes ofídicos y los géneros asociados, en el departamento de Nariño, 2008-2017.

En la figura 5, se muestra la georreferenciación de los casos ocurridos en Nariño y los géneros de ofidios implicados en los mismos de acuerdo con la información consignada en las fichas de notificación, y en el cuadro 4, se presenta un listado de posibles especies de serpientes que potencialmente ocasionan o podrían ocasionar accidentes ofídicos teniendo en cuenta los registros en las bases de datos descritas en la sección de materiales y métodos.

**Cuadro 4:** Familias, géneros y posibles especies asociadas con el accidente ofídico en el departamento de Nariño

Familias y subfamilias	Géneros	Especies
		
Viperidae	*Bothrops*	*Bothrops asper*
		*Bothrops* aff. *atrox*
		*Bothrops punctatus*
		*Bothrops ayerbei*
	*Lachesis*	*Lachesis muta*
	*Crotalus*	*Crotalus durissus cumanensis*
Elapidae	*Micrurus*	Micrurus mipartitus*
		Micrurus ancoralis*
		*Micrurus* aff. *spixii*
		*Micrurus* aff. *lemniscatus*
		Micrurus clarki*
		Micrurus dumerilii*
	*Hydrophis*	*Hydrophis platurus*
Colubridae Dipsadinae	*Leptodeira*	*Leptodeira annulata*
		*Leptodeira septentrionalis*
	*Oxyrhopus*	*Oxyrhopus petola*
Colubrinae	*Oxybelis*	*Oxybelis brevirostris*
	*Leptophis*	*Leptophis ahaetulla*
	*Stenorrhina*	*Stenorrhina degenhardtii*

* Pitalúa, *et al*. (2018) determinó la distribución potencial de estas especies en Nariño a partir de modelamiento de nicho y algunas de estas especies cuentan con ejemplares depositados y registrados en la colección zoológica de la Universidad de Nariño (datos no publicados).

A partir de las fichas de notificación confirmadas y en las que el agente causal fue reconocido, se extrajo la información sobre la sintomatología local y sistémica de los pacientes, y se identificaron los signos y síntomas más frecuentes. Esta información fue comparada en paralelo con descripciones previamente publicadas de las manifestaciones clínicas (locales y sistémicas) y las alteraciones paraclínicas características de cada tipo de envenenamiento (botrópico, crotálico, lachésico, elapídico) ^12^^,^^36^ (cuadros 5 y 6), con el objetivo de verificar la congruencia entre la especie que ocasionó el accidente y el cuadro clínico que presentaron los pacientes. 

**Cuadro 5:** Principales síntomas locales asociados con los géneros de serpientes de importancia médica en el departamento de Nariño, 2008-2017

Manifestaciones clínicas locales	*Bothrops*		*Lachesis*		*Micrurus*		*Hydrophis*		*Crotalus*		Colubridae*	
Frecuencias	fi	fi/n	fi	fi/n	fi	fi/n	fi	fi/n	fi	fi/n	fi	fi/n
Absceso	24	0,05	_	_	_	_	_	_	_	_	_	_
Dolor	421	0,87	13	0,76	21	0,75	8	1	29	0,91	12	0,86
Edema	398	0,82	10	0,59	18	0,64	5	0,63	22	0,69	9	0,64
Equimosis	53	0,11	2	0,12	1	0,036	_	_	4	0,13	1	0,07
Eritema	189	0,39	6	0,35	11	0,39	3	0,38	14	0,44	4	0,29
Flictenas	36	0,07	3	0,18	_	_	_	_	3	0,09	_	_
Hematoma	34	0,07	_	_	2	0,07	_	_	1	0,03	_	_
Celulitis	122	0,25	4	0,24	7	0,25	3	0,38	9	0,28	2	0,14
Fasceítis	8	0,02	_	_	_	_	_	_	_	_	_	_
Mionecrosis	5	0,01	1	0,06	_	_	_	_	_	_	_	_
Alteraciones en la circulación/perfusión	16	0,03	_	_	_	_	_	_	_	_	1	0,07
Gangrena		X		_		_		_		_		X
Hemorragias		X		_		_		_		_		X
Linfadenitis regional		X		_		_		_		_		_
Necrosis tisular	20	0,04	1	0,06	_	_	_	_	1	0,03	_	_
Paresia local y regional	_	_	_	_	_	_	_	_	5	0,16	_	_
Parestesias	68	0,14	3	0,18	9	0,32	_	_	_	_	2	0,14
Sangrado		X		_		_		_		_		_
Adenopatía		X		_		_		_		_		_
Calambres		X		_		_		_		_		_
Prurito		X		_		_		_		_		_
Rubor		_		_		_		X		_		_
Dolo lumbar		_		_		_		_		_		X

fi: frecuencia; fi/n: frecuencia relativa donde n es el número de casos por género n: número de casos por género; *: No se informa el género; -: datos no reportados; X: se omitieron las frecuencias de estos síntomas locales clasificados en el ítem ‘otros’ en la ficha de notificación, ya que podrían representar datos sesgados en comparación con los que se listan en la ficha. No se incluyen síntomas sin registro en el cuadro clínico de los pacientes (hipotermia).

**Cuadro 6:** Principales síntomas sistémicos asociados con los géneros de serpientes de importancia médica en el departamento de Nariño, 2008-2017

Manifestaciones clínicas sistémicas	*Bothrops*		*Lachesis*		*Micrurus*		*Hydrophis*		*Crotalus*		Colubridae*	
	fi	fi/n	fi	fi/n	fi	fi/n	fi	fi/n	fi	fi/n	fi	fi/n

Alteraciones de la visión	25	0,05	1	0,06	3	0,11	_	_	2	0,063	_	_
Bradicardia	18	0,04	_	_	1	0,04	_	_	_	_	_	_
Cefalea		X		_		_		_		_		_
Diarrea	9	0,02		_		_		_		_		_
Dificultad para hablar	13	0,03	1	0,06	_	_	1	0,13	2	0,06	_	_
Disfagia	2	0,004	_	_	_	_	2	0,25	_	_	_	_
Epistaxis	7	0,01	_	_	_	_	_	_	_	_	_	_
Escalofríos		X		_		_		_		_		_
Falla renal		X		_		_		_		_		_
Facies neurotóxica	9	0,02		_	1	0,04		_		_		_
Fiebre		X		_		_		X		X		X
Gingivorragia	46	0,10	_	_	2	0,07	_	_	2	0,063	_	_
Hemoptisis		X		_		_		_		_		_
Hipotensión	37	0,08	_	_	_	_	_	_	1	0,031	_	_
Mareo		X		_		_		_		_		_
Convulsiones		X		_		_		_		_		_
Disminución del llenado capilar		X		_		_		_		_		_
Dificultad respiratoria		X		_		_		_		_		_
Hematuria	24	0,05		_		_		_	2	0,06	1	0,07
Alteraciones de la coagulación		X		_		_		_		_		_
Calor		X		_		_		X		_		_
Diaforesis		X		_		_		_		X		_
Hipertermia		_		_		X		_		_		_
Dolor abdominal	38	0,08	3	0,18	2	0,07	_	_	_	_	_	_
Alteración sensorial	9	0,02	_	_	1	0,04	_	_	_	_	_	_
Debilidad muscular	66	0,14	3	0,18	5	0,18	_	_	4	0,13	_	_
Hematoquexia	4	0,01	_	_	_	_	_	_	_	_	_	_
Midriasis		_		_		_		_		_		X
Náuseas	135	0,28	3	0,18	14	0,5		_		_	3	0,21
Parestesias		X		_		_		_		_		_
Ptosis palpebral	1	0,002	_	_	_	_	1	0,13	_	_	_	_
Choque hipovolémico	10	0,021	_	_	1	0,04	_	_	_	_	_	_
Choque séptico	8	0,02	1	0,06	1	0,04	_	_	1	0,03	1	0,07
Sialorrea	17	0,04	_	_	_	_	_	_	1	0,03	_	_
Síndrome compartimental		X		_		_		_		_		_
Vértigo	44	0,09	1	0,06	4	0,14	_	_	2	0,06	1	0,07
Vómito	89	0,18	2	0,12	3	0,11	_	_	5	0,16	1	0,07
Choque anafiláctico		X		_		_		_		_		_
Taquipnea		X		_		_		_		_		_
Melanemesis		X		_		_		_		_		_
Alteración de la conciencia		X		_		_		_		_		_
Coluria		X		_		_		_		_		_
Paro cardiorrespiratorio		X		_		_		_		_		_
Hipertensión		X		_		_		_		_		_
Coagulación intravascular diseminada	11	0,02	_	_	_	_	_	_	_	_	_	_
Hemorragia subaracnoidea	3	0,01	_	_	_	_	_	_	_	_	_	_
Edema cerebral	3	0,01	_	_	_	_	_	_	_	_	_	_
Falla ventilatoria	9	0,02	_	_	1	0,04	_	_	_	_	_	_
Coma	3	0,01	_	_	_	_	_	_	_	_	_	_
Anemia aguda	18	0,04	_	_	1	0,04	_	_	2	0,06	_	_
Infección respiratoria aguda	9	0,02	1	0,06	1	0,04	_	_	_	_	_	_
Oliguria	13	0,03	_	_	_	_	_	_	_	_	_	_
Cianosis	9	0,02	_	_	1	0,04	_	_	_	_	_	_
Hematemesis	27	0,06	_	_	_	_	1	0,13	_	_	_	_

fi: frecuencia; fi/n: frecuencia relativa donde n es el número de casos por género n: número de casos por género; *: no se informan géneros relacionados; -: datos no reportados; X: se omitieron las frecuencias de estos síntomas sistémicos clasificados en el ítem ‘otros’ en la ficha de notificación, ya que podrían representar datos sesgados en comparación con los que se listan en la ficha. No se incluyen síntomas sin registro en el cuadro clínico (diplopía, estrabismo divergente, falla orgánica multisistémica, facies miasténica, melenas, mialgias, oftalmoplejía, papiledema, rabdomiólisis, arritmia cardíaca, paro respiratorio).

Aquellos que sufrieron envenenamiento por el género *Bothrops*, generalmente, presentan síntomas locales como abscesos, dolor, edema, celulitis, fascitis, perfusión, equimosis, flictenas, gangrena, hemorragia local, eritema, linfadenitis regional, necrosis tisular, parestesias y hematomas, y manifestaciones sistémicas, como diarrea, epistaxis, emesis, anemia aguda, alteraciones en la visión, sialorrea, cefalea, gingivorragia, hemoptisis, melena, náuseas, hematemesis, necrosis, choque hipovolémico y séptico, debilidad muscular y hematuria.

Por otra parte, el cuadro clínico del envenenamiento elapídico se caracteriza por escasos síntomas locales (dolor, edema, parestesia y celulitis) y manifestaciones sistémicas, como alteraciones de la visión, facies neurotóxica, náuseas, sialorrea, vómito, vértigo, cianosis, fallas respiratorias, choque hipovolémico y séptico.

En los cuadros 5 y 6, se listan las principales manifestaciones clínicas de los envenenamientos producidos por los géneros de serpientes responsables del ofidismo en Nariño y su ocurrencia con base en el número de casos totales por género.

### Manejo del accidente ofídico por parte de la entidad de salud

Los centros hospitalarios provistos de suero antiofídico son: Hospital San Andrés E.S.E., en Tumaco; Hospital Sagrado Corazón de Jesús E.S.E., en El Charco; Hospital Lorencita Villegas de Santos E.S.E., en Samaniego, y Fundación Hospital San Pedro y Hospital Universitario Departamental de Nariño, en San Juan de Pasto. En estos centros de salud, se atiende el mayor número de accidentes ofídicos que suceden en Nariño y en departamentos cercanos, como Cauca y Putumayo.

De acuerdo con la sintomatología local y sistémica, la mayoría de los casos fueron leves (n=647); de estos, el 63,37 % de los pacientes fueron tratados con suero antiofídico.

El cuadro 7 muestra los tipos de sueros antiofídicos utilizados en el manejo de casos con grado de envenenamiento leve, moderado y grave, durante el periodo comprendido entre 2008 y 2017. El suero polivalente fue utilizado en 711 casos (64,05 %), el monovalente fue empleado en el tratamiento de 54 casos (4,86 %) y el suero antiofídico anticoral fue usado en 4 casos (0,52 %). En promedio, los accidentes botrópicos leves se trataron con tres viales (rango de 1 a 12), los moderados, con cinco viales (rango de 1 a 21), y los graves, con siete viales (rango de 1 a 20). En promedio, los accidentes crotálicos leves se trataron con tres viales (rango de 1 a 8), los moderados, con cinco viales (rango de 1 a 12), y los graves, con siete viales (rango de 7 a 8).

**Cuadro 7:** Gravedad del accidente ofídico, administración y tipo de suero antiofídico utilizado, en el departamento de Nariño, 2008-2017

Variable	Característica	n	Porcentaje	Casos tratados con	Porcentaje	Tipo de suero		
		
suero antiofídico	1	2	3
				
								
Gravedad del accidente	Leve	647	58,29	410	63,37	378	29	3
	Moderado	352	31,71	281	79,83	258	22	1
	Grave	96	8,65	75	78,13	72	3	0
	No envenenamiento	15	1,35	4*	26,67	3	0	0

*Solo se registra el tipo de suero en 3 de los 4 casos. Los tipos de suero (1, polivalente (botrópico, lachésico, crotálico), 2, monovalente, 3, anti-coral) se diferencian de acuerdo con la información consignada en las fichas de notificación del accidente ofídico, versiones 2008 a 2017.

## Discusión

### Frecuencia e incidencia de los accidentes ofídicos

Según los estudios retrospectivos sobre la epidemiología del accidente ofídico, en otros departamentos de Colombia se presentaron frecuencias menores a las reportadas para Nariño (111 casos anuales), entre 2008 y 2017; por ejemplo, en el departamento del Cauca, se reportó una frecuencia de 42 casos por año entre 2000 y 2008, y en los departamentos de Magdalena y Sucre, se reportaron promedios de 97 y 18 casos, durante un periodo de 5 y 6 años, respectivamente ^2^^,^^37^. No obstante, la incidencia del ofidismo en Nariño fue menor respecto a los mismos departamentos en ese periodo ^23^^-^^25^^,^^38^^-^^44^. Estas diferencias se explican por las variaciones de la densidad poblacional en los departamentos durante los años de estudio, así como por los distintos periodos analizados y el número total de casos acumulados.

Los municipios más afectados por el accidente ofídico en el departamento de Nariño fueron aquellos localizados en las zonas bajas y costeras, lo que concuerda con los resultados expuestos por Cuéllar, *et al*. ^2^. Estos municipios se caracterizan por presentar ecosistemas que favorecen la presencia de serpientes, por ejemplo, el municipio de San Andrés de Tumaco situado sobre la llanura pacífica, una región lluviosa que incluye zonas de bosque pluvial premontano y bosque húmedo tropical y abarca desde el nivel del mar hasta los 600 metros de altura ^45^.

El aumento del número de casos y la incidencia anuales del accidente ofídico en Nariño entre 2008 y 2013, podría explicarse por el incremento en los registros desde que la ficha única de notificación de accidente ofídico y el protocolo de vigilancia del evento fueron implementados en el año 2005, y divulgados a nivel nacional a comienzos del 2007 ^42^, lo cual logró cada vez una mayor preocupación por notificar el evento de parte de los centros de salud.

Entre 2014 y 2015, el número de casos fue relativamente constante tanto en Nariño como en Colombia, lo cual refleja una notificación más constante del evento ^23^^,^^44^. El descenso de casos entre 2016 y 2017 en Nariño, en contraste con el incremento de casos en el país (4.704 y 4.978, respectivamente), puede deberse a un hecho real o a cuestiones de subregistro; pese a que este fenómeno ha disminuido paulatinamente, no ha desaparecido por completo y es expuesto de forma preocupante por varios autores ^12^^,^^28^^,^^37^.

Desde el año 2009 hasta la semana epidemiológica 39 de 2011, se reportaron 96 fallecimientos ante el Sivigila, con una mortalidad promedio de 0,7 % casos por cada 1’000.000 de habitantes colombianos, y los departamentos de Antioquia, Bolívar, Córdoba y Nariño fueron los más afectados ^30^^,^^46^. En 2013, año en el que se reportó el mayor número de accidentes ofídicos en Nariño, se notificaron 28 muertes de pacientes en Córdoba, Nariño y Sucre, con una letalidad del 0,64 % y una mortalidad de 0,59 casos por 1’000.000 de habitantes para el país; ese mismo año, Nariño ocupó el quinto lugar a nivel nacional y la lista fue encabezada por el municipio de Tumaco ^46^.

Es preocupante el registro de defunciones para el departamento. Los registros de 27 defunciones entre el 2008 y 2017 demuestran que los pacientes notificaron el accidente relativamente rápido ante un centro hospitalario; sin embargo, de acuerdo con las descripciones clínicas, la mayoría de los casos fueron graves, lo cual dificultó el tratamiento oportuno. Además, en el 47,48 % de los casos no se reporta el género o la especie de la serpiente agresora, lo que dificulta la selección del tipo de suero (polivalente o monovalente) por parte del personal médico si no se tiene suficiente conocimiento de las manifestaciones clínicas propias de cada envenenamiento; esto podría conducir al uso incorrecto del suero y, como consecuencia, a no obtener el efecto neutralizador esperado; por ejemplo, uno de los casos de muerte fue atribuido a una especie del género *Micrurus* y el paciente fue tratado con suero polivalente (botrópico, lachésico y crotálico), desconociéndose las razones de dicha decisión. Por otro lado, el número de ampollas que se utilizó en estos envenenamientos graves, 10 viales, no era el recomendado por el protocolo de manejo ^47^ y solamente en el 38,46 % de los casos se cumplió con lo establecido.

Con respecto a la frecuencia estacional de los accidentes ofídicos, se observó que son más frecuentes trimestralmente entre mayo y julio, y alcanzan una máxima frecuencia e incidencia en el mes de julio. Lo anterior concuerda con los resultados de los informes finales de ofidismo presentados por el Instituto Nacional de Salud durante los últimos 10 años ^23^^-^^25^^,^^38^^-^^44^ y con los análisis descriptivos realizados en los departamentos de Sucre ^37^ y Magdalena ^2^. Las condiciones climatológicas en Nariño entre los meses de abril a julio, caracterizadas por fuertes precipitaciones (2.250 mm) en los núcleos y sectores lluviosos del departamento ^48^^,^^49^, podrían favorecer el incremento de los accidentes ofídicos debido a la dificultad de localizar serpientes en el momento de eliminar malezas en los cultivos durante las actividades agrícolas, al tránsito por zonas inundadas y a caminar por senderos abiertos o trochas. Entre los meses de octubre a enero, el número de encuentros fortuitos con serpientes posiblemente disminuye, ya que las precipitaciones se reducen considerablemente (1.000-1.750 mm), incluso produciendo sequias ^48^^,^^49^.

Boadas, *et al*., informaron que la frecuencia de casos de ofidismo en Venezuela fue significativamente mayor en la estación de alta pluviosidad (junio a septiembre) y, menor, en la de mediana pluviosidad (octubre a enero) ^50^. En Costa Rica, el análisis del accidente ofídico en relación con las temporadas de mayores precipitaciones, muestra una asociación negativa con la frecuencia de casos ocurridos en el sur del Valle del Pacífico (región húmeda) y una asociación positiva en el norte del Valle del Pacífico (región seca). Estos patrones implican que las variaciones meteorológicas en los diferentes países guardan relación con el comportamiento del evento ^2^ por ser factores determinantes en la oferta de presas para las serpientes y en su fenología reproductiva ^51^.

A nivel regional, el trimestre de mayor incidencia (mayo-julio) se relaciona estrechamente con las temporadas de las cosechas de café, palma de aceite y otros cultivos, incluyendo los ilícitos; sus plantaciones, al generar importantes cantidades de hojarasca heterogénea (necromasa), se convierten en nichos apropiados para la proliferación de serpientes ^52^ y, por ende, en un riesgo potencial de sufrir accidentes ofídicos.

El sexo más afectado fue el masculino, aspecto que es igual a lo observado en trabajos similares ^2^^,^^14^^,^^23^^-^^25^^,^^37^^,^^42^^-^^44^. Es probable que la mayor frecuencia de accidentes en la población masculina se deba a las actividades de trabajo de campo (agricultura) culturalmente asignadas a los hombres que, por tal razón, se ven obligados a caminar por senderos abiertos o trochas y a permanecer en terrenos donde habitan las serpientes. Independientemente de lo anterior, en este trabajo se reporta que las labores de tipo doméstico, actividades realizadas por mujeres, también están asociadas a accidentes ofídicos.

Dentro de los municipios, las zonas donde predominaron los accidentes ofídicos fueron las rurales, las dispersas o ambas (74,50 %); estos resultados concuerdan con los informes anuales del Instituto Nacional de Salud, lo cual confirma lo expuesto por la tipología establecida en el protocolo de vigilancia ^31^, donde se afirma que en las zonas rurales o áreas dispersas se incrementa el riesgo de accidente ofídico ^14^^,^^37^.

Por otro lado, para la región pacífica, las etnias víctimas de ofidismo fueron principalmente: negro, mulato y afrocolombiano, y el rango de edad más afectado fue el de 16 a 30 años, resultados que concuerdan con lo reportado en la mayoría de las investigaciones sobre epidemiología del accidente ofídico ^2^^,^^14^^,^^23^^-^^25^^,^^37^^,^^43^^,^^44^^,^^46^^,^^48^^,^^50^^,^^53^^,^^54^.

Los motivos por los cuales existe mayor frecuencia de ofidismo en esta población etaria, están relacionados con el hecho de ser esta región del país una de las más productivas en agricultura y deforestación sistemática; por otra parte, existen muchos jóvenes trabajadores y menores de edad que habitan en zonas rurales o que acompañan a los adultos a sus jornadas laborales en el campo, por la carencia de educación en materia de prevención del accidente ofídico, encuentros accidentales o, simplemente, la curiosidad e imprudencia al enfrentarse a una serpiente ^50^.

### Serpientes responsables del ofidismo

En el periodo de 2008 a 2017, el género *Bothrops* ocasionó el mayor número (43,60 %) de accidentes ofídicos en el departamento. La información suministrada por las fichas técnicas en estos casos evidencia la presencia de huellas de colmillos en el sitio de la mordedura, y sintomatología local y sistémica propia del accidente botrópico. Este género ha sido reportado como el principal agente causal de ofidismo en diferentes trabajos a nivel nacional e internacional ^2^^,^^14^^,^^37^^,^^50^.

El amplio rango de distribución de algunas de las especies (por ejemplo, *Bothrops asper*), su relativa abundancia y la capacidad de habitar en lugares crípticos o con cierto grado de intervención, son las principales razones por las cuales este género genera el mayor número de envenenamientos. En Nariño, este género se distribuye principalmente sobre la vertiente oeste de la Cordillera Occidental, en la región pacífica, lo que explicaría que el 78,47 % de los casos se presenten en dicha zona (cuadro 1, figuras 1 y 5) ^53^.

El agente causal del accidente no fue identificado en 47,48 % de los casos, posiblemente porque la serpiente no se capturó (67,03 %) (cuadro 3), o por ausencia de registro fotográfico, muerte y deterioro de la serpiente, desconocimiento de su nombre común y fallas en la descripción del espécimen, lo cual obstaculiza su reconocimiento taxonómico.

La integración de la georreferenciación de los casos, el agente causal y las especies de serpientes reportadas en Nariño por las diferentes bases de datos, permitieron identificar los municipios donde existe mayores probabilidades de sufrir un accidente ofídico y reconocer especies potencialmente peligrosas en el departamento (figuras 4 y 5). Además, teniendo en cuenta la sintomatología local y sistémica y el hecho de que el 78,74 % de estos casos evidencian huellas de colmillos en el lugar de la mordedura (cuadro 3), es probable que las serpientes responsables de estos accidentes sean principalmente de dentición solenoglifa (familia Viperidae) por presentar colmillos grandes, retráctiles y localizados en la parte anterior de la maxila; mientras que las serpientes proteroglifas y opistoglifas tienen colmillos pequeños, situados hacia la parte media-anterior y posterior de la maxila, respectivamente, lo cual dificulta un poco más la mordedura ^12^. Entre los géneros que podrían estar involucrados en dichos accidentes, están *Bothrops*, *Bothriopsis*, *Bothriechis*, *Bothrocophias*, *Lachesis* y *Porthidium* (figura 4 , cuadro 4).

De los cinco géneros potencialmente peligrosos en el departamento: *Bothrops*, *Crotalus*, *Lachesis*, *Micrurus* e *Hydrophis*, este último fue el que presentó menor incidencia (0,72 %), hecho que concuerda con lo expuesto por Ayerbe en el 2008 y 2009 ^12^ quien denominó a este accidente como “el más raro de todos” y solo reportó tres accidentes ocasionados por la especie *Hydrophis platurus*, anteriormente denominada *Pelamis platurus*. En este estudio, se reportan ocho casos durante los 10 años. Su poca frecuencia se relaciona con la historia natural de esta especie de hábitos migratorios. Aunque es la serpiente con mayor rango de distribución en el mundo, es marina, se aproxima a la costa Pacífica (donde se han reportado los accidentes) entre enero y mayo; se caracteriza por poseer colmillos muy pequeños en la parte anterior de la maxila (dentición proteroglifa), lo cual dificulta la inoculación de veneno ^3^^,^^12^.

Resulta desconcertante el hecho de que el género *Crotalus* representara el 2,88 % de los casos, ya que no existen registros de la especie en Nariño. *Crotalus durissus cumanensis* es la subespecie del género con distribución en Colombia. Se encuentra en el norte de la costa Atlántica, el valle del río Magdalena y la Orinoquía, su distribución más al sur incluye al municipio de Garzón en el departamento del Huila y los municipios de Inzá y Páez en el departamento del Cauca ^1^^,^^3^^,^^12^^,59,60)^; habita un rango altitudinal que abarca desde el nivel del mar hasta cerca de los 2.500 m, en el centro del país ^3^.

Los accidentes ofídicos ocasionados por esta especie son catalogados como raros por Ayerbe (2008, 2009) ya que tienen una incidencia menor del 1 % ^12^. Los casos notificados en Nariño podrían explicarse como resultado de un reconocimiento equívoco por parte del paciente o del personal médico encargado del registro, posiblemente por ausencia de fotografía o captura del espécimen, pues en el 68,75 % de estos casos, aunque se identificó a la serpiente, no existe evidencia de dicha identificación.

Es difícil suponer que, con la característica física (el cascabel) que presentan estas serpientes se puedan confundir con otras, pero existe la posibilidad de confundirla con serpientes que emiten sonidos similares con las escamas de la cola ^55^. Una explicación alternativa sería que, ocasionalmente, las cascabeles son transportadas por los llamados ‘culebreros’ hacia regiones del país donde normalmente no habitan con fines de entretenimiento, porque se tiene la creencia de que el cascabel de esta especie ‘cura el cáncer’ ^2^^,^^3^, sin descartar el tráfico de fauna.

Infortunadamente, esta explicación es difícil de sustentar porque, en las fichas de notificación de los casos por esta especie, la actividad registrada durante el accidente fue: actividad agrícola (40,63 %), oficios domésticos (18,75 %), actividad no descrita bajo la categoría ‘Otros’, y otras, como recreación, recolección de desechos y caminar por senderos o trochas. Por lo anterior, los casos atribuidos a *C. durissus cumanensis* no pueden ser homologados, pues la clínica reportada no es la típica del envenenamiento crotálico en Suramérica, con una o dos excepciones (cuadros 5 y 6).

Será relevante hacerles seguimiento a las nuevas notificaciones de casos por esta especie, con el fin de confirmar la veracidad de los datos; además, capacitar al personal de salud que los registra para que reconozcan las especies potencialmente peligrosas en Nariño, a partir de la información suministrada por los pacientes. De esta manera, se relacionarían mejor las manifestaciones clínicas del envenenamiento con el agente causal, y se lograría brindar tratamientos más efectivos.

### Manejo del accidente ofídico por profesionales de la salud

La mayoría de los envenenamientos fueron tratados con el suero polivalente disponible en Colombia, capaz de neutralizar el veneno de varios géneros de serpientes de la misma familia (*Bothrops*, *Crotalus* y *Lachesis*), asociados al accidente ofídico en el departamento ^56^^,^^57^.

Si el personal médico tiene información sobre el agente agresor y sabe que la mayoría de los accidentes en el país son ocasionados por el género *Bothrops*, tratará a los pacientes con este tipo de suero. No obstante, también es probable que, por desconocer el género o la especie que causó el accidente, se emplee suero antibotrópico por ser el de mayor espectro.

Generalmente, las dosis de suero antiofídico empleadas para el envenenamiento botrópico en Nariño, son las recomendadas en el protocolo de manejo; en este se establece que los casos leves deben manejarse con dos ampollas de las producidas por el Instituto Nacional de Salud, los moderados, con cuatro ampollas, y los graves, con seis ampollas. Sin embargo, no se emplean las recomendadas para el accidente crotálico, en el cual se deben aplicar seis ampollas en casos leves, ocho en el moderado y 10 en el grave. El número de ampollas se duplica en cada caso, cuando se usa el suero producido por los Laboratorios *Probiol*, S. A. ^47^.

También, se encontraron reportes de casos graves en los cuales no se usó el número de ampollas requeridas y, además, que se usó suero antiofídico en 26,67 % de los pacientes registrados como ‘sin envenenamiento’ (cuadro 7); esta situación es similar a la que ocurría en el departamento de Santander a finales del siglo pasado (González VG. Seroterapia y tratamiento del accidente ofídico en el departamento de Santander. Primer Simposio Colombiano de Toxinología. Medellín: 1998. p. 149-55).

Durante los 10 años analizados, se empleó el suero monovalente en 54 casos, leves, moderados o graves, lo que sugiere que el personal médico tenía mayor certeza del agente causal, porque el ejemplar fue capturado, fotografiado o reconocido por las descripciones de la víctima. Sin embargo, los centros de salud han informado que, cuando la serpiente es reconocida y no existe provisión del suero apropiado, se utiliza indistintamente el polivalente ^2^^,^^12^^,^^46^. Esta situación es grave desde el punto de vista clínico, ya que se incrementa el riesgo de desencadenar un cuadro de alergia al suero.

Por otra parte, en los centros hospitalarios de primer nivel que no cuentan con el equipo necesario, la falta de actualización de las fichas de notificación obligatorias, su incorrecto manejo y la dificultad para diagnosticar alteraciones como el edema cerebral o la hemorragia subaracnoidea, entre otras, favorecen el subregistro de los síntomas locales y sistémicos, y esto se refleja en inconsistencias entre el cuadro clínico y el tratamiento administrado.

En este sentido, Cuéllar-Gordo, *et al*. ^2^, Zambrano ^46^ y Ayerbe (Ayerbe S. Seroterapia y tratamiento del accidente ofídico en el departamento del Cauca. Primer Simposio Colombiano de Toxinología. Medellín, 1998. p. 149-55), manifiestan que prevalecen fallas en el cumplimiento del protocolo de manejo del accidente ofídico, pues, en algunos casos, no existe coherencia entre la identificación del género de la serpiente, el cuadro clínico y la clasificación de la gravedad del accidente, y la dosis y el tipo de suero antiofídico administrado.

A partir de este análisis retrospectivo, se concluye que la provincia biogeográfica más afectada es la Pacífica, y que el municipio de San Andrés de Tumaco presenta el mayor número de casos, por lo cual su población tiene mayor riesgo de sufrir un accidente ofídico dada la diversidad de especies que alberga. El género *Bothrops* es el responsable de la mayoría de los accidentes en Nariño, los cuales ocurren generalmente en las áreas rurales, afectan principalmente a la población masculina y son más frecuentes en el mes julio.

En este contexto, es necesario alertar a las entidades de salud pública, con el fin de mejorar el aprovisionamiento de suero, principalmente, en esta región y, así, lograr un tratamiento oportuno. El análisis del manejo de los accidentes en nuestro departamento deja en evidencia la necesidad de capacitar al personal médico en el reconocimiento del cuadro clínico de cada tipo de envenenamiento y la atención de los pacientes cumpliendo con los protocolos dispuestos para este fin.

## References

[1] Campbell J, Lamar W (2004). The venomous reptiles of the western hemisphere. The Herptile.

[2] Cuéllar-Gordo LC, Amador-Orozco B, Olivares-Goenaga G, Borré-Ortiz YM, PinedoOtálvaro J (2015). Comportamiento epidemiológico del accidente ofídico en el departamento del Magdalena, Colombia (2009-2013). Revista Ciencias de la Salud.

[3] Lynch JD (2012). El contexto de las serpientes de Colombia con un análisis de las amenazas en contra de su conservación. Revista de la Academia Colombiana de Ciencias Exactas, Físicas y Naturales.

[4] Ministerio de la Proteccion Social (2004). Resolución número 2934 de 2004. Diario Oficial No. 45.672 de septiembre 15 de 2004.

[5] Duque JF, Sánchez A, Fierro L, Garzón S, Castaño RS (2007). Venenos de serpientes y moléculas antiveneno. Revista de la Academia Colombiana de Ciencias Exactas, Físicas y Naturales.

[6] Gutiérrez JM (2002). Comprendiendo los venenos de serpientes: 50 años de investigaciones en América Latina. Rev Biol Trop.

[7] Boldrini-França J, Cologna CT, Pucca MB, Bordon K de CF, Amorim FG, Anjolette FAP (2017). Minor snake venom proteins: Structure, function and potential applications. Biochim Biophys Acta.

[8] Chippaux JP, Williams V, White J (1991). Snake venom variability: Methods of study, results and interpretation. Toxicon.

[9] Alape-Girón A, Sanz L, Escolano J, Flores-Díaz M, Madrigal M, Sasa M (2008). Snake venomics of the lancehead pitviper Bothrops asper: Geographic, individual, and ontogenetic variations. Proteome.

[10] Franco F, Cardoso JL França FO, Wen FH Malaque CM, Haddad editors (2003). Origem e diversidade das serpentes. Animais peçonhentos no Brasil: biologia, clínica e terapêutica dos acidentes.

[11] Uetz P The Reptile Database.

[12] Ayerbe-González S, Ordóñez CA, Ferrada R, Buitrago R (2009). Ofidismo en Colombia, enfoque, diagnóstico y tratamiento. Cuidados intensivos y trauma.

[13] Carrasco PA, Mattoni CI, Leynaud GC, Scrocchi GJ (2012). Morphology, phylogeny and taxonomy of South American bothropoid pitvipers (Serpentes, Viperidae). Zool Scr.

[14] Wallach V, Williams KL, Boundy J (2014). Snakes of the world. A catalogue of living and extinct species.

[15] Gutiérrez JM, Williams D, Fan HW, Warrell DA (2010). Snakebite envenoming from a global perspective: Towards an integrated approach. Toxicon.

[16] Organización Mundial de la Salud Mordeduras de serpiente.

[17] Valderrama R (2010). Animales ponzoñosos de Latinoamérica. Biomédica.

[18] Bravo CA (2015). Modelo matemático epidemiológico para estimar el sub-reporte de envenenamientos por serpientes en Colombia.

[19] Rodríguez-Vargas AL (2012). Comportamiento general de los accidentes provocados por animales venenosos en Colombia, 2006-2010. Rev Salud Pública.

[20] Hernández-Camacho J, Hurtado-Guerra A, Ortiz-Quijano R, Walschburger T, Halffter G (1992). Unidades biogeográficas de Colombia. La diversidad biológica de Iberoamérica.

[21] Delgado A, Ruiz S, Arévalo L, Castillo G, Viles N (2008). Plan de acción en biodiversidad del departamento de Nariño 2006-2030.

[22] Lynch JD, Angarita-Sierra T, Ruiz-Gómez FJ (2014). Programa Nacional para la Conservación de las Serpientes presentes en Colombia.

[23] León LJ (2015). Informe final del evento accidente ofidico, Colombia.

[24] León LJ (2016). Informe del evento accidente ofidico, Colombia, 2016.

[25] Rojas MA (2017). Accidente ofídico Colombia, 2017. Sivigila.

[26] Instituto Nacional de Salud (2018). Semana epidemológica 52 de 2018.

[27] Gómez JP (2011). Accidente por animales ponzoñosos y venenosos: su impacto en la salud ocupacional en Colombia. Revista Facultad Nacional de Salud Pública.

[28] Charry-Restrepo H (2006). Epidemiología del accidente ofídico en Colombia. Temas de Toxinología.

[29] Noguera-Urbano EA (2016). Mastozoología en Nariño y algunos comentarios sobre la biogeografía de la región. Rev Ciencias.

[30] Ministerio de Salud Sistema de Vigilancia en Salud Pública.

[31] Walteros D, Paredes A (2014). Protocolo de Vigilancia en Salud Pública. Accidente ofídico.

[32] Departamento Administrativo Nacional de Estadística (2005). Censo general.

[33] Sistema de Información sobre Biodiversidad de Colombia.

[34] Global Biodiversity Information Facility. Colombia.

[35] Instituto Geográfico Agustín Codazzi Geoportal.

[36] Sarmiento-Acuña K (2012). Aspectos biomédicos del accidente ofídico. Universitas Médica.

[37] Márquez MA, Gómez GM (2015). Accidente ofídico en el departamento de Sucre, Colombia. NOVA.

[38] Heredia D (2008). Comportamiento del accidente ofídico en Colombia, 2008.

[39] Heredia D (2009). Informe anual de accidente ofidico, 2009.

[40] Heredia D, Paredes AE (2010). Informe final del evento accidente ofídico en Colombia hasta el décimo tercer periodo epidemiológico 2010.

[41] Paredes AE (2011). Informe del evento accidente ofídico, hasta el periodo epidemiológico 13 de 2011.

[42] Paredes AE (2012). Informe del evento accidente ofídico final año 2012.

[43] Paredes AE (2014). Informe del evento accidente ofidico, Colombia, 2013.

[44] León LJ (2014). Informe final del evento accidente ofidico Colombia, año 2014.

[45] Tejada C, Otero L, Castro L, Afanador F, Devis A, Solano J (2003). Aportes al entendimiento de la Bahía de Tumaco. Entorno oceanográfico, costero y de riesgos.

[46] Zambrano ÁM (2012). Accidente ofídico como evento de interés en salud pública en Colombia: aportes al diseño de estrategias de gestión.

[47] Peña LM, Zuluaga AF (2017). Protocolo manejo del paciente intoxicado.

[48] Molano J, Batista J (1967). Calendario climatológico aeronáutico colombiano.

[49] Herrera MT, Beltrán G, Rincón AV, Gómez NF (2016). Nariño. Características geográficas.

[50] Boadas J, Matos M, Bónoli S, Borges A, Vásquez-Suárez A, Serano L (2012). Reportes epidemiológicos. Perfil eco-epidemiológico de los accidentes por ofidios en Monagas, Venezuela (2002-2006). Boletín Malariol y Salud Ambient.

[51] Chaves LF, Chuang T, Sasa M, Gutiérrez JM (2015). Snakebites are associated with poverty, weather fluctuations and El Niño. Sci Adv.

[52] Villavicencio-Enríquez L (2012). Producción, pérdida de peso y tasas de descomposición de hojarasca en cafetales tradicional y rústico, y selva mediana, en Veracruz, México. Revista Chapingo serie Ciencias Forestales y del Ambiente.

[53] Bolaños R (1982). Las serpientes venenosas de centroamérica y el problema del ofidismo. Primera parte. Aspectos zoológicos, epidemiológicos y biomédicos. Rev Cost Cienc Méd.

[54] Guerrero-Bermúdez FJ (2010). Caracterización epidemiológica de los accidentes ofídicos, en pacientes pediátricos, Cartagena de Indias 2006-2007. Revista Ciencias Biomédicas.

[55] Young BA (2003). Snake bioacoustics: Toward a richer understanding of the behavioral ecology of snakes. Q Rev Biol.

[56] Instituto Nacional de Salud Suero antiofídico polivalente.

[57] Laboratorios Probiol Suero antiofidico.

